# The Application and Optimization of HIPEF Technology in the Processing of Juice from Strawberries Harvested at Two Stages of Ripeness

**DOI:** 10.3390/foods11141997

**Published:** 2022-07-06

**Authors:** Anica Bebek Markovinović, Predrag Putnik, Višnja Stulić, Luka Batur, Boris Duralija, Branimir Pavlić, Tomislava Vukušić Pavičić, Zoran Herceg, Danijela Bursać Kovačević

**Affiliations:** 1 Faculty of Food Technology and Biotechnology, University of Zagreb, Pierottijeva 6, 10000 Zagreb, Croatia; abebekmarkovinovic@pbf.hr (A.B.M.); luka.batur@alumni.unizg.hr (L.B.); tvukusic@pbf.hr (T.V.P.); zherceg@pbf.hr (Z.H.); dbursac@pbf.hr (D.B.K.); 2 Department of Food Technology, University North, Trg dr. Žarka Dolinara 1, 48000 Koprivnica, Croatia; 3 Department of Dietetics, University Hospital Centre Zagreb, Mije Kišpatića 12, 10000 Zagreb, Croatia; 4 Department of Pomology, Division of Horticulture and Landscape Architecture, Faculty of Agriculture, University of Zagreb, Svetošimunska Cesta 25, 10000 Zagreb, Croatia; bduralija@agr.hr; 5 Faculty of Technology, University of Novi Sad, Blvd. Cara Lazara 1, 21000 Novi Sad, Serbia; bpavlic@uns.ac.rs

**Keywords:** high intensity pulsed electric field (HIPEF), bioactive compounds, strawberry juice, maturity, storage

## Abstract

The aim of this study was to investigate the influence of high intensity pulsed electric field (HIPEF) technology on the stability of total phenols, anthocyanins, hydroxycinnamic acids, flavonols, and condensed tannins in strawberry juices (*Fragaria x ananassa* Duch. cv. ‘Albion’) with different ripening stages (75% and 100%) and stored at +4 °C for 7 days. The HIPEF parameters studied were: (i) electric field strength (40 and 50 kV cm^−1^), (ii) frequency (100 and 200 Hz), and (iii) treatment duration (3 and 6 min). Of the HIPEF parameters studied, electric field strength and frequency had a statistically significant effect on the content of all phenolic compounds. Treatment duration showed no statistically significant effects on phenolic compounds except for flavonols and condensed tannins. Storage had a positive effect on the stability of most of the phenolic compounds, with the exception of flavonols. Optimization of HIPEF processing showed that strawberry samples at both ripeness levels were suitable for HIPEF treatment to obtain functional fruit juices with a high content of polyphenols.

## 1. Introduction

Given the global pandemic of chronic diseases, many of which are directly related to obesity, immense efforts are being made to highlight the importance of higher consumption of fruit and vegetables as a preventive measure [1,2]. In this case, foods with distinct functional properties have attracted particular attention because of their promising health benefits [3,4]. Strawberry (*Fragaria x ananassa* Duch.) is widely recognized as a fruit with functional properties, mainly due to its high concentration of bioactive compounds such as polyphenols and/or vitamins [1,5,6]. Environmental conditions, cultivation technique and harvest time can have a great influence on the fruit quality of cv. ‘Albion’ [7]. There is ample evidence of the antioxidant, antihypertensive, antihyperlipidemic, and antiproliferative effects of strawberries [1]. Among the numerous bioactive compounds, ascorbic acid, ellagitannins, and anthocyanins are the most potent in yielding certain health benefits [1]. Considering the short harvest season, but also due to consumer preferences, strawberries are processed into various products, of which jams, purees, and juices are the most common [1]. For example, Zhao et al. investigated the antioxidant and antibacterial potential of functional strawberry juice inoculated with a starter culture of lactic acid bacteria (LAB) and yeasts [8]. A similar methodology was used by Cataldo et al. in their work in which strawberry juice was inoculated with *Levilactobacillus brevis* to study the immunomodulatory effect of gamma-aminobutyric acid on rodent cell cultures [9]. In addition to processing into juice, studies have also shown the potential of processing strawberries into dry powders [10]. A considerable number of studies, including human intervention studies and cell lines using fresh or frozen fruits, extracts, purees, etc., some of which are covered in the 2014 review by Basu et al., illustrated the great potential and, what is now evident, the great interest in using strawberries as valuable health promoters [1].

However, it is important to minimize the loss of bioactive compounds of strawberries during processing to preserve the “functional character” of the final product. Additionally, the selection of the appropriate cultivar and degree of ripeness is of great importance when developing a product with a high content of bioactive compounds from strawberries. For example, Mazur et al. evaluated the suitability of three different strawberry cultivars (‘Blink’, ‘Polka’, and ‘Senga Sengana’) at three stages of ripeness (‘nearly ripe’, ‘ripe’, and ‘fully ripe’) for the production of jam [11]. Although there were differences in total phenolic content (TP) among different cultivars, still they were no statistically different nor did they differ significantly with maturation. Consequently, no significant differences were found in the TP content of the jams produced, although jams made from ‘fully ripped’ strawberries had the highest TP content. Nevertheless, this study suggests that fully ripe fruits are more suitable for processing to better preserve product color during storage. However, Bebek Markovinović et al. showed that the strawberry cultivar ‘Albion’ harvested at 75% ripeness had a significantly higher content of TP and was also suitable for processing, although the juice produced from 100% ripe fruit had a higher TP content, which indeed was not statistically significant from that which was produced from 75% ripe fruit [12]. Interestingly, the authors addressed the potential of strawberry by-products, which also showed a high content of bioactive compounds. However, the strength of this study lies in the fact that the selection of partially ripened fruits can be extremely useful for the industry, as strawberries are highly susceptible to damage during transportation and storage, which consequently has a negative impact on the sensory quality of the final product. Therefore, less ripe fruits may be more suitable for industrial purposes, as they are more “resistant” to logistical factors.

Several factors must be considered when processing strawberries into functional products. First of all, the strawberry itself is an excellent substrate for bacterial growth due to its high-water content and the amount of sugar present [13]. These factors must be considered especially for designing of strawberry juice, where the aforementioned issues are even more intensified. Attention should be paid not only to the biochemical composition and its preservation, but also to microbiological safety. To date, thermal pasteurization is one of the safest processing methods, but at the same time it results in a deterioration of the nutritional and bioactive contents of the final product, in this case juices [13,14]. Thermal processing is also responsible for color changes in juices, such as browning, that can make the product unacceptable to consumers seeking a fresh and attractive red color of the juice [15]. In view of this, novel non-thermal methods of food preservation such as high-intensity pulsed electric field processing (HIPEF) are being developed and tested to meet the high demand for functional products with nutritional and sensory properties similar to those of fresh fruits [16,17,18]. The results of a previous study [12] have shown that both ripening grades (75% and 100%) of strawberries (*Fragaria x ananassa* Duch., cultivar ‘Albion’) are suitable for the production of functional juices. Therefore, the aim of this work was to study the influence of different parameters of HIPEF technology on the content of bioactive compounds in strawberry juices produced at different degrees of ripeness and stored for 7 days at +4 °C. Based on the obtained results, the optimal parameters for HIPEF treatment that ensure the best preservation of bioactive compounds in the juices were also calculated.

## 2. Materials and Methods

### 2.1. Chemicals and Standards

Sodium carbonate, anhydrous (99.5–100.5%), hydrochloric acid (37%, *w*/*w*), sulfuric acid (96%, p.a.), and formic acid (98%, p.a.) were obtained from Lach-ner (Neratovice, Czech Republic). HPLC 99% pure methanol purchased from Honeywell (Paris, France) was used as the extraction solvent. For spectrophotometric analysis of total phenols used Folin–Ciocalteu reagent procured from Fisher Scientific UK (Loughborough, UK). Ethanol (96% pure) was procured from Gram-mol (Zagreb, Croatia). Quercetin (95%) and gallic acid standard (97.5–102.5%) were purchased from Acros Organics (Guangzhou, China) and Sigma-Aldrich (St. Louis, MO, USA). Potassium chloride (99.0–100.5%), vanillin (99%), sodium acetate anhydride (99%), and chlorogenic acid (min. 95%) were obtained from Thermo Fisher (Kandel, Germany).

### 2.2. Strawberry Juice Preparation

Strawberry fruits (*Fragaria x ananassa* Duch., cv. ‘Albion’) were harvested in 2021 at the company Jagodar HB in Donja Lomnica, Zagreb County (Croatia). The strawberries were harvested at two different stages of ripeness: (i) at the technological ripening stage, where the fruits are 75% red (F1), and (ii) at the full ripening stage, where the fruits are 100% red (F2). Immediately after harvest, strawberries were delivered to the laboratory, where they were removed from the stems, washed with tap water and dried with cellulose. Then, juices (J1 and J2) were prepared from the strawberries of the appropriate degree of ripeness (F1 and F2) by cold pressing (Kuvings B6000 Slow Juicer, VerVita d.o.o., Zagreb, Croatia). The juices produced in this way were subjected to HIPEF treatment whose process parameters are described in Section 2.3. All juices were filled in sterile glass bottles and hermetically sealed.

### 2.3. High-Intensity Pulsed Electric Field (HIPEF) Processing of Strawberry Juice Samples

**Figure 1:**
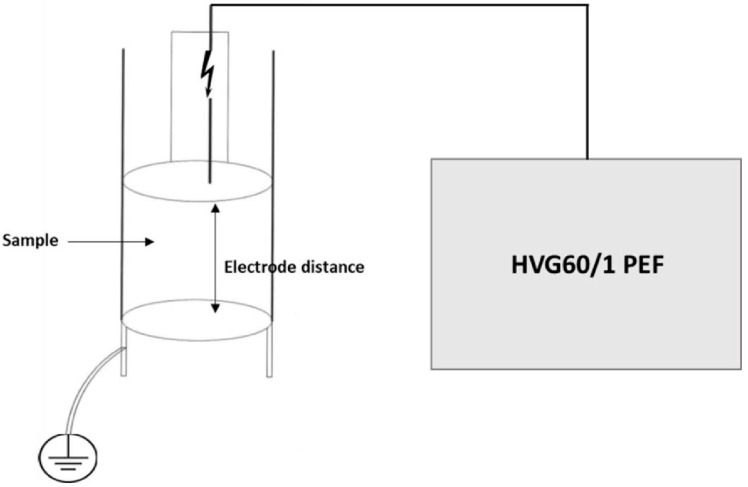
Schematic depiction of HIPEF reactor.

The HIPEF device used in this research was HVG60/1 HIPEF (Impel d.o.o., Zagreb, Croatia). HIPEF treatments were performed by placing the strawberry juice samples in the treatment chamber at room temperature. The treatment chamber configuration was two stainless steel electrodes (Figure 1) with a diameter of 68 mm, a gap distance of 1 cm, and a volume of 100 mL. Treatments were conducted using square wave pulses, duration of 0.5 µs at the frequencies of 100 and 200 Hz. The applied electric field strength ranges were 40 and 50 kV cm^−1^. Total treatment time was 3 and 6 min, and the total applied energy ranged from 1.425 × 10^2^–6.750 × 10^2^ kJ L^−1^.

The control samples were untreated juices, while the HIPEF treatment conditions were as follows: electric field 40 kV cm^−1^ and 50 kV cm^−1^, frequency 100 Hz and 200 Hz with pulse duration 3 min and 6 min, according to design of the experiment (Table 1). A batch of juices was analyzed immediately after HIPEF processing, and another batch of juices were stored at 4 °C for 7 days. The stored juice samples were evaluated during the storage period to determine the evolution of the quality indices related to the physiochemical aspects and the stability of the bioactive compounds.

### 2.4. Characterization of Untreated and HIPEF-Treated Strawberry Juices

#### 2.4.1. Determination of pH and Soluble Solids Content (SSC)

pH was determined with a Mettler Toledo FiveEasy pH meter (Mettler-Toledo GmbH, Greifensee, Switzerland) previously calibrated with commercial buffer solutions at pH 4.0 and 7.0. SSC (Brix%) was determined with a digital refractometer ATAGO Pal-3 (ATAGO Co., Tokyo, Japan). Duplicate measurements were performed for each sample.

#### 2.4.2. Extraction of Bioactive Compounds

According to a modified protocol from the literature [14], the extractions of biologically active compounds from prepared strawberry juices (J1 and J2) were performed. A total of 5 mL of the sample and 20 mL of 1% formic acid in 80% methanol (*v*/*v*) were mixed and vortexed for 1 min. Then, the reaction mixture was extracted at 50 °C for 15 min in an ultrasonic bath (DT 514 H Sonorex Digitec 13.5 L, Bandelin electronic, Berlin, Germany) and centrifuged at 10,000 rpm/10 min (Thermo Scientific™, Megafuge™ 16R, Kalkberg, Germany). The supernatant was then filtered and made up to 25 mL with extraction solvent in a volumetric flask. All extracts were prepared in duplicates. The extracts were stored at −18 °C under inert gas atmosphere until analysis.

#### 2.4.3. Determination of Total Phenolic Content (TPC)

Total phenolic content was measured using the Folin–Ciocalteau modified spectrophotometric assay described in the literature [19]. Volume of 400 µL of the extract was mixed with 400 µL of the FC reagent (previously diluted 5 -fold with distilled water) and 4 mL of a 7.5% sodium carbonate solution (*w*/*v*). The reaction mixture was allowed to stand at room temperature for 20 min and the absorbance of the colored reaction was measured at 725 nm using a spectrophotometer (LLG-uniSPEC 2 Spectrophotometer, Buch & Holm, Meckenheim, Germany). Duplicate measurements were performed for each sample. The TPC in the extracts was calculated using a standard calibration curve generated with different concentrations of gallic acid (10–250 mg L^−1^). Results were expressed as mg gallic acid equivalent (GAE) per 100 g or 100 mL of sample.

#### 2.4.4. Determination of Total Monomeric Anthocyanins (ANT)

Anthocyanins were determined by the spectrophotometric differential pH method [20]. A total of 1 mL of the extract was mixed with 4 mL of 0.4 M sodium acetate buffer (pH 4.5) and separately with 4 mL of 0.025 M potassium chloride buffer (pH = 1.0). The reaction mixture was allowed to stand at room temperature for 20 min and absorbance was measured at 520 and 700 nm using a spectrophotometer (LLG-uniSPEC 2 Spectrophotometer, Buch & Holm, Meckenheim, Germany). Deionized water was used as a blank. Duplicate measurements were performed for each sample. The concentration of monomeric anthocyanins in the sample was expressed as pelargonidin-3-glucoside equivalent (Pg-3-G) (mg L^−1^) according to calculation as described in the literature [21].

#### 2.4.5. Determination of Total Hydroxycinnamic Acids (HCA)

HCA was determined by a modified spectrophotometric method [22]. A volume of 250 µL of the extract was mixed with 250 µL of solution 1 (1 g L^−1^ solution of HCl dissolved in 96% ethanol) and 4.55 mL of solution 2 (2 g L^−1^ HCl dissolved in distilled water). The reaction was vortexed for 10 s and then allowed to react in the dark at room temperature for 30 min. The absorbance of the reaction was measured at 320 nm in a spectrophotometer (LLG-uniSPEC 2 Spectrophotometer, Buch & Holm, Meckenheim, Germany). For the blank sample, the extraction solvent was used instead of the extract, and the rest of the procedure remained the same as for the sample. Duplicated measurements were performed for each sample. The HCA content was calculated using a calibration curve generated with different concentrations of chlorogenic acid (10–600 mg L^−1^). The results were expressed as mg chlorogenic acid equivalent (CAE) per 100 g or 100 mL of the sample.

#### 2.4.6. Determination of Total Flavonols (FL)

FL were determined by modified spectrophotometric method [22]. A volume of 250 µL of the extract was mixed with 250 µL of solution 1 (1 g L^−1^ solution of HCl dissolved in 96% ethanol) and 4.55 mL of solution 2 (2 g L^−1^ HCl dissolved in distilled water). The reaction was vortexed for 10 s and then allowed to react in the dark at room temperature for 30 min. The absorbance of the reaction was measured at 360 nm in a spectrophotometer (LLG-uniSPEC 2 Spectrophotometer, Buch & Holm, Meckenheim, Germany). For the blank sample, the extraction solvent was used instead of the extract, and the rest of the procedure remained the same as for the sample. Duplicated measurements were made for each sample. The FL in the extracts were calculated from a calibration curve obtained with different concentrations of quercetin solution (10–600 mg L^−1^). Results were expressed as mg quercetin equivalent (QE) per 100 g or 100 mL of the sample.

#### 2.4.7. Determination of Condensed Tannins (CT)

CT was determined by a modified spectrophotometric method [23]. A volume of 2.5 mL of reagent 1 (1% vanillin solution in methanol) was mixed with reagent 2 (2.5 mL of 25% H_2_SO_4_ solution in methanol) and 1 mL of extract. The reaction mixture was stirred with a vortex for 1 min and then allowed to react at room temperature for 10 min. Then, the absorbances were measured at 500 nm in a spectrophotometer (LLG-uniSPEC 2 Spectrophotometer, Buch & Holm, Meckenheim, Germany). A blank sample was prepared in the same way, except that the extraction solvent was used instead of the sample. Duplicated measurements were made for each sample. A catechin standard solution (10–120 mg L^−1^) was used to prepare the calibration curve, and the results were expressed as mg catechin equivalent (CA) per 100 g or 100 mL of the sample.

### 2.5. Statistical Analysis

Experiments were designed as full factorial randomized experimental design. Dependent variables for multivariate analysis were: pH; SSC—soluble solids content (%); TPC—total phenolic compounds (mg 100 mL^−1^); ANT—anthocyanins (mg 100 mL^−1^); HCA—hydroxycinnamic acids (mg 100 mL^−^^1^); FL—flavonols (mg 100 mL^−1^), and CT—condensed tannins (mg 100 mL^−1^). Independent variables were: sample maturity (75%, 100%); storage (0 and 7 days). HIPEF settings evaluated were: electric field strength (40 and 50 kV cm^−1^), frequency (100 Hz, 200 Hz), and treatment time (3 and 6 min). The significance levels for all tests were α ≤ 0.05. Analyses were performed with IBM SPSS Statistics (v.24). The RSM optimization analysis was done with STATGRAPHICS Centurion XVIII. Occurrence of overparameterization was tested with variance inflation factors that were all lower than acceptable value (V.I.F. ≤ 4) so that all models are precise and with very good predictive power.

## 3. Results and Discussion

### 3.1. Changes of the Physicochemical Parameters in Untreated and HIPEF-Treated Strawberry Juices during Storage

Of the physicochemical parameters in untreated strawberry juices during storage, pH and SSC were studied (Table 2). The average value for SSC in untreated juices was 7.09%, while in HIPEF-treated samples it was 6.87%. Untreated strawberry juices had a pH of 3.41 and HIPEF-treated samples had a pH of 3.47. As can be seen, the J2 strawberry juices had higher SSC and pH values than the J1 juices, which can be attributed to the higher ripeness of the strawberries used for J2 production. Since SSC value is strongly correlated with fruit ripeness [12], it was expected that a higher SSC value would be observed in riper fruits. In addition, as the fruit ripens, total acidity decreases, which is reflected in an increase in pH [24,25].

A storage period of 7 days affected the reduction of SSC in untreated strawberry juices. Similarly, previous work reported that SSC may gradually decrease during cold storage [26], which may be due to the denaturation of enzymes [27]. The pH of the untreated juice samples remained stable during storage. However, Aaby et al. found that the pH of strawberry purée decreased during storage, which can be attributed to the formation of small amounts of organic acids as a result of initial microbial contamination [28]. The decrease in pH during storage can also be attributed to the stabilization of pH due to low temperature during storage [23].

Similar to the untreated samples, HIPEF samples with higher maturity (J2) were determined to have higher SSC and pH (Table 3). The effect of storage on physicochemical parameters for HIPEF-treated strawberry juices was identical to that of untreated juices, with SSC decreasing and pH remaining unchanged during storage. Therefore, the treated samples had lower SSC (6.78 ± 0.01%) than at the beginning of storage (6.97 ± 0.01%). These results are in agreement with those of Mtaou et al. who also found a statistically significant decrease in the SSC value of date juice treated with HIPEF (35 kV cm^−^^1^, 100 Hz, 1000 ms) during 5 weeks of storage at 4–5 °C [29].

On the other hand, these results are in contrast with the results of Geveke et al. who found an opposite trend in SSC values of strawberry puree during 24 weeks of accelerated storage at 40 °C [30]. These discrepancies in the results could be explained by different storage conditions, or more precisely, in this case, different storage temperatures. Namely, there is a possibility that due to the higher storage temperature, there was a greater evaporation of water from the product and consequently an increase in the SSC value compared to storing of the product at lower temperatures.

Increasing the electric field strength from 40 kV cm^−^^1^ to 50 kV cm^−^^1^ resulted in a significant increase in SSC (6.82 ± 0.01% vs. 6.92 ± 0.01%), which can be explained by the HIPEF phenomenon of electrophoresis that enhanced the release of cell biomaterial by degrading the cellular structures in the colloid system of the juice [31,32]. However, frequency and treatment duration had no effect on SSC in the juice samples.

Storage, electric field strength, and duration of treatment did not affect the pH of the samples. Similarly, Odriozola Serrano et al. showed that HIPEF treatment (35 kV cm^−^^1^, 100 Hz and 1700 µs) did not produce significant differences in pH change before (3.39 ± 0.05) and after HIPEF treatment (3.38 ± 0.03) [33]. The obtained results are in agreement with the results of Geveke et al. who studied the influence of HIPEF technology on the physicochemical parameters of strawberry puree during accelerated storage for a period of 24 weeks. In their study, the pH values of HIPEF-treated strawberry puree did not change during storage [30].

### 3.2. The Changes in the Stability of Bioactive Compounds of Untreated Strawberry Juices during Storage

Strawberry juice is a valuable source of biologically active compounds, as confirmed by the results of this study, in which the total phenolic content (TPC) in untreated samples was 79.78 ± 1.25 mg GAE 100 mL^−^^1^ (Table 4). Considering the subgroups of phenolic compounds, condensed tannins (CT) were the most abundant (75.82%), followed by anthocyanins (ANT) (33.63%), hydroxycinnamic acids (HCA) (14.09%), and flavonols (FL) (2.54%). Although juice J1 from the less ripe strawberries had higher TPC content than juice J2 from fully ripe strawberries (85.19 vs. 74.39 mg GAE 100 mL^−^^1^), all other bioactive compounds were detected at higher concentrations in strawberry juices from fully ripe strawberries (J2). Since there is no evidence in the literature that strawberries from different stages of ripeness were processed into juice, it can be assumed that these results are consistent with previous findings for fresh fruit at different stages of ripeness [24].

The ANT concentration in J2 was 1.25-fold higher than in J1, which is consistent with previous results in strawberries at different stages of ripening [34]. The significantly higher HCA concentration in J2 than in J1 was explained by Pradas et al. [34] in terms of phenolic acid accumulation, since these compounds serve as precursors for the different branches of the phenylpropanoid pathways and phenolic compound metabolism [35]. In addition, the different contents of FL in strawberries from different ripening stages seemed to correlate with variations in cultivars, as there is evidence in the literature that the content of FL in strawberries increases or decreases with ripening depending on the cultivar type [34].

During storage, the content of TPC, ANT, HCA, and CT generally increased significantly by about 20–36%, regardless of the maturity stage at harvest. After 7 days at 4 °C, the TPC content in strawberry juices (96.02 mg 100 mL^−1^) was 1.5 times higher (*p* ≤ 0.05) than at the beginning of storage (63.55 mg 100 mL^−1^). In strawberry fruit (*Fragaria x ananassa* Duch. cv. ‘Gariguette’), an increase in TPC was also observed during 2 days of storage at 10 °C [36].

### 3.3. The Influence of HIPEF Processing on the Stability of Bioactive Compounds in Strawberry Juices during Storage

The effect of HIPEF treatment, ripeness, and storage parameters on the bioactive compounds of the HIPEF-treated samples is shown in Table 5. The average content of TPC in the treated samples was 89.49 ± 0.50 mg GAE 100 mL^−^^1^. As maturity increased from 75% to 100%, the TPC content decreased by 15% (96.91 mg 100 mL^−^^1^ vs. 82.08 mg 100 mL^−^^1^). These results are most likely related to the fact that phenolic compounds are synthesized in the superficial part of the fruit tissue, and, as the fruit ripens, their mass increases, so that the proportion of phenolic compounds is higher in unripe fruit than in ripe fruit [37]. On the other hand, TPC levels increased by 15% during prolonged storage from the 1st to the 7th day of storage. Odriozola Serrano et al. studied the effects of HIPEF technology (35 kV cm^−^^1^, 100 Hz and 1700 µs) on the quality of strawberry juice during 56 days of storage at +4 °C. The data show an increase in TPC up to 21 days of storage, after which the TPC begins to decrease [33]. With increasing electric field strength, the TPC increased significantly, which can be explained by electroporation and facilitated leakage of biomaterial due to cell wall damages [38,39,40]. These results are also in agreement with the results of a study by Siddeeg et al. who investigated the effect of different electric field strengths (1, 2 and 3 kV cm^−^^1^, 10 Hz, 100 µs) on the bioactive components of date palm fruit [41]. Here, a decrease when the frequency was increased from 100 Hz (90.69 mg/100 mL^−^^1^) to 200 Hz (88.31 mg 100 mL^−^^1^) of almost 3% was observed, while the treatment time had no effect on the content of TPC.

Since the color of the juice is the first visual impression that consumers perceive and determines their perception of the product, the content of anthocyanins in HIPEF-treated juice and the effects of HIPEF parameters on anthocyanin content are very important. The average content of anthocyanins in the treated samples was 28.49 ± 0.13 mg 100 mL^−^^1^ (Table 5). As expected, samples with higher ripeness had higher contents of anthocyanins, thus samples with 100% ripeness (37.98 mg 100 mL^−^^1^) had almost 100% more anthocyanins than samples with 75% ripeness (19.01 mg 100 mL^−^^1^). With longer storage, the content of anthocyanins was higher (29.63 mg 100 mL^−^^1^) than at the beginning of storage (27.35 mg 100 mL^−^^1^).

When considering the influence of electric field strength (40 vs. 50 kV cm^−^^1^), the results showed that higher electric field strength resulted in higher anthocyanin content, likely due to increased extraction of anthocyanins from the matrix by HIPEF treatment [42]. A previous report showed that the contents of anthocyanins in strawberry juices after HIPEF treatment with an electric field strength between 20 and 35 kV cm^−^^1^ for up to 2000 μs, varied only slightly and was 96 to 100% of the original content, depending on the processing intensity [33]. Similarly, the results of this study showed that the content of anthocyanins in untreated strawberry juices was 26.83 mg 100 mL^−^^1^, while this concentration in HIPEF-treated juices was 28.60 mg 100 mL^−^^1^. The content of anthocyanins decreased with increasing frequency, as a slight decrease of 2% was observed when the frequency was doubled from 100 Hz (28.79 mg 100 mL^−^^1^) to 200 Hz (28.19 mg 100 mL^−^^1^). Odriozola Serrano et al. showed that a range between 100 and 250 Hz in HIPEF treatment of strawberry juices positively affected the stability of the initial anthocyanin content. However, the effect of frequency on anthocyanin retention strongly depended on the pulse width used [43]. The treatment time did not change the anthocyanin content.

Next, the contents of hydroxycinnamic acids were examined, which averaged 12.36 ± 0.09 mg 100 mL^−^^1^ in the treated samples (Table 5). As with the other polyphenols, the hydroxycinnamic acid content increased with maturity (10.62 mg 100 mL^−^^1^ for 75% maturity vs. 14.10 mg 100 mL^−^^1^ for 100% maturity). Similar to before, longer storage time also increased the hydroxycinnamic acid contents in the samples, by almost 43%. Galani et al. found that cold storage of fruits and vegetables had positive effect on the stability of phenolic acids, which was due to the accumulation of sugars, released after HIPEF treatment that could serve as substrates for their synthesis [44]. As observed with other polyphenol groups, the content of hydroxycinnamic acids increased with increasing electric field strength. Although no data comparing the effects of different electric field strengths on hydroxycinnamic acid content were found in the literature, changes in hydroxycinnamic acid content could be due to the degradation of more complex polyphenolic structures in HIPEF treatments [45]. Another emerging pattern similar to all studied polyphenols seem to be inverse relation among increased frequency and content of polyphenols in the samples. This is evident from the evaluation of hydroxycinnamic acids, whose content decreased slightly when the frequency was doubled from 100 Hz (12.60 ± 0.12 mg 100 mL^−^^1^) to 200 Hz (12.12 ± 0.12 mg 100 mL^−^^1^), or for about 4%. As with the other polyphenols, treatment duration had no effect on hydroxycinnamic acid content.

In addition, the average value of flavonols was 2.15 ± 0.03 mg 100 mL^−^^1^ in the data set of treatment samples (Table 5). As with other polyphenols, higher maturity yields more flavonols, as samples with 100% maturity (2.48 ± 0.04 mg 100 mL^−^^1^) had 37% more flavonols than samples with 75% maturity (1.81 ± 0.04 mg 100 mL^−^^1^; Table 4). In contrast to all other polyphenols, the content of flavonols decreased three-fold with prolonged storage, as their initial content before storage was 3.29 ± 0.04 mg 100 mL^−^^1^, while after 7 days it was only 0.99 ± 0.04 mg 100 mL^−^^1^. This was in agreement with the results of Odriozola Serrano et al. who found a 44% retention of quercetin during storage of HIPEF-treated strawberry juice (56 days at 4 °C) [33]. As with all other polyphenols, the same trend with increasing electric field strength was observed for flavonols as well. Here, a field strength of 50 kV cm^−^^1^ (2.38 ± 0.04 mg 100 mL^−^^1^) resulted in a 1.3-fold higher contents of flavonols than the strength of 40 kV cm^−^^1^ (1.91 ± 0.04 mg 100 mL^−^^1^). As before, a pattern for field frequency was observed for flavonols as for other polyphenols. Consequently, the field at 100 Hz frequency had 2.29 ± 0.04 mg 100 mL^−^^1^ flavonols (or 13% more) than 2.00 ± 0.04 mg 100 mL^−^^1^ for 200 Hz. In contrast to all other polyphenolic groups, the duration of HIPEF treatment decreased the flavonols content from 2.29 ± 0.04 mg 100 mL^−^^1^ (3 min) to 1.99 ± 0.04 mg 100 mL^−^^1^ (6 min) or by 13%. Odriozola Serrano et al. found a low flavonol content in HIPEF-treated strawberry juice (35 kV cm^−^^1^, 100 Hz and 1700 µs) compared to phenolic acids and anthocyanins, which is in agreement with our results [33].

The last group of polyphenols evaluated were condensed tannins, which accounted for the majority of the total the polyphenol content (or 67.49 ± 0.14 mg 100 mL^−^^1^) in the treated samples, and also represented the largest group of compounds in these experiments (Table 5). Similar to anthocyanins and hydroxycinnamic acids, the content of condensed tannins increased with increasing ripeness of strawberries. Interestingly, the content of all polyphenols increased during storage, except flavonols, whose concentration decreased (Table 5). Flavonols decreased by almost 70% after 7 days of storage, while condensed tannin content increased by 46%. This is somewhat expected since flavonols are known to condense to tannins [46], which was probably captured in the experiments and data. As with all other polyphenols, the content of condensed tannins increased with increasing electric field strength, which makes sense in the context of electroporosity and its well-documented relationship with field strength [40]. Although there are no data in the literature on the effects of different electric field strengths on condensed tannin contents, a study conducted by Manzoor et al. showed a significant increase in condensed tannin content in almond extracts compared to untreated samples [47].

Increasing the frequency, degraded 4% of the condensed tannin content, similar to what was previously observed for all other polyphenols. This strongly suggests that the electric field frequency has an optimal value that must be determined to obtain the maximum nutritional value, e.g., as measured by the polyphenol content in this case [48]. Similarly, for the HIPEF treatment time, it can be observed that when the time was doubled, 3% of polyphenols were degraded, from which we can also conclude that these parameters of the HIPEF treatment are sufficiently effective at a shorter treatment time (3 min). Moreover, in almost all cases, the treatment time did not show a significant effect on the content of bioactives (with the exception of flavonols and condensed tannins). This could perhaps be explained by the fact that the HIPEF process achieves its maximum extraction effect with a shorter treatment time. Increasing the treatment time from 3 min to 6 min had a negative effect on the flavonols and condensed tannins content, indicating possible degradation of these compounds at longer treatment time.

### 3.4. Comparison of the Stability of Bioactive Compounds in Treated vs. Untreated Strawberry Juices during Storage

When considering the stability of the bioactive compounds, it can be seen that the untreated samples had the same stability patterns (Table 4 and Table 5). However, the HIPEF treatment resulted in slightly higher values of all the bioactive compounds determined, so this technology is not only an alternative to pasteurization, but can also be successfully used to better maintain the stability of bioactive compounds in strawberry juices. Overall, HIPEF treatment increased bioactive compound levels by an average of 8.41% in all treated strawberry juices, regardless of ripeness and processing parameters.

However, an interesting observation was made in the data set regarding changes in polyphenols with increasing maturity and storage. In the untreated J2 juice samples, condensed tannins increased by 15% (at 75% to 100% maturity). This can be considered a normal condensation rate in the samples due to the increasing ripening of the strawberries. However, in the samples treated with HIPEF with the same ripening variation, this value was halved (8% condensation). The situation was similar for the storage and condensation of tannins. Here, the normal condensation rate for prolonged storage (from 0 to 7 days) was 94%. When the samples were treated with HIPEF, the condensation rate was halved to 46% for the same storage period.

Similarly, anthocyanins increased 25% in controls throughout storage, while they decreased three-fold in HIPEF-treated samples (only 8% increase for the same storage period). Increased degradation of cyanidin-3-glucoside under the influence of HIPEF in aqueous methanol medium was found by Zhang et al. while the increase of field strength and treatment duration had no significant effect on the food system [49]. This may indicate the influence of abundance on the decrease of anthocyanins, which was shown in our results by a decrease in the content of several groups of biologically active compounds. Accordingly, the content of total phenolics increased by 51% with storage (0–7 days) in the controls, while this rate was 15% in the HIPEF samples for the same storage parameters (in other words, HIPEF decreased the increase of all polyphenols threefold).

It is likely that anthocyanins and condensed tannins defined the above relations in the samples (i.e., they were mainly responsible for the observed changes in total phenolic content). For instance, observed data strongly suggested that HIPEF in some way halved the spontaneous increase and condensation of polyphenols (potentially important for personal customization of foods or synthesis of nutraceuticals if combined with data for particular bioavailability). This occurrence was potentially associated with previously mentioned increase in HIPEF frequency, which showed a tendency to decrease polyphenolic concentrations. A possible explanation could be sought in the differently charged species generated by HIPEF, which are capable of shifting electrostatic stability toward the degradation of tannins and anthocyanins when generated at sufficient concentrations. This makes even more sense considering that these two groups of polyphenols were the most represented in the samples. Another important conclusion from the data is the need to determine the optimal value for the aforementioned HIPEF treatment parameters.

### 3.5. Optimization of HIPEF Processing Parameters for Strawberry Juice Treatment

HIPEF technology belongs to the group of non-thermal technologies, which means that it operates at room temperature, or below or slightly above room temperature, thus enabling the preservation of heat-sensitive bioactive compounds [50]. Table 6. shows the average values of the HIPEF settings, where temperature can be highlighted as the most important factor influencing the quality of the final product. The average temperature of the treated samples was T2 = 22.93 ± 0.40 °C (room temperature) and no significant temperature differences were observed before and after HIPEF treatment (ΔT = 1.68 ± 1.00 °C), which is consistent with other literature data [14,16]. Since no statistically significant temperature variations were observed during HIPEF treatment, this confirms that temperature did not affect the stability of bioactive compounds in HIPEF-treated samples.

The average voltage in the samples was 44.91 ± 0.11 kV with an average expenditure of 3.42 ± 0.01 mA and power of 154.88 ± 0.51 W. Since voltage, current, and power are related to the electric field strength and frequency (excluding voltage) parameters, it is not surprising that there are statistically significant variations in these values. Voltage (kV) or electric field strength (kV cm^−1^) has a direct effect on the electroporation of membrane cells in a plant, which translates into elevated release of bioactive compounds. A stronger electric field with a longer treatment time leads to irreversible electroporation of tissue cells and consequently tissue damages, which is the basis of how HIPEF technology works [51].

**Figure 2:**
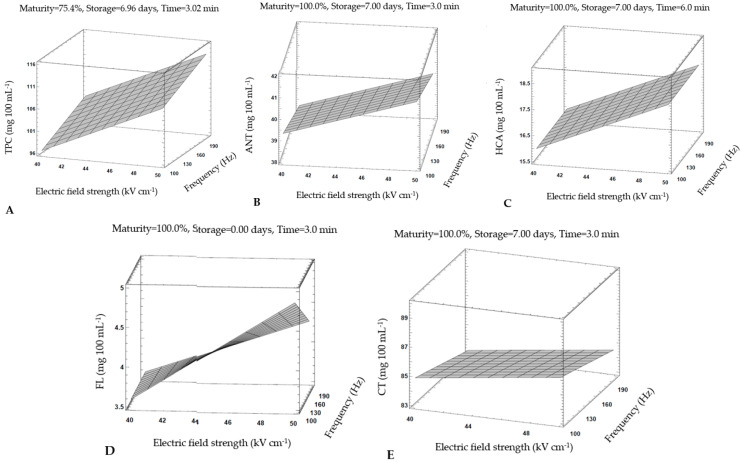
Response surface plots with optimal HIPEF parameters for all bioactive compounds in strawberry juices: (**A**) total phenolic compounds; (**B**) anthocyanins; (**C**) hydroxycinnamic acids; (**D**) flavonols; (**E**) condensed tannins.

In order to find out which parameters of HIPEF treatment are most favorable for preservation of bioactive compounds in strawberry juice, process optimization was performed. Figure 2 shows response surface plots with the optimal HIPEF parameters for each group of bioactive compounds. The highest content of total polyphenols can be obtained at settings slightly different for each polyphenol group. For example, 113.75 mg 100 mL^−1^ of total polyphenols can be best obtained after almost 7 days of storage at lower ripeness (near 75% ripeness) with HIPEF settings of 49.90 kV cm^−1^ at a frequency of 199.74 Hz and a treatment time of 3.02 min (Table 7).

This was similar to other individual polyphenol groups, except that all other polyphenols had the highest concentrations at lower frequency (100 Hz) and higher maturity (100%). These results suggested that strawberry juices of both ripeness levels are suitable for HIPEF treatment, and depending on which bioactive constituents are to be better preserved in the final product, the strawberry raw material for juice production could be selected based on its ripeness level.

All increases in polyphenol content favored longer storage (except flavonols) and shorter treatment times (except hydroxycinnamic acids). Regarding the storage and its positive effects on bioactive compound contents (except flavonols), a possible explanation could be in the subsequent extraction or leakage of bioactive compounds from damaged tissue cells. Odriozola Serrano et al. performed a kinetic study on anthocyanins, vitamin C and antioxidant activity of strawberry juice treated with HIPEF technology (20, 25, 30, and 35 kV cm^−1^ during 100, 300, 600, 1000, 1500 and 2000 μs at 232 Hz). The results of their studies agree with ours, confirming that anthocyanins are better preserved with a shorter treatment time and a stronger electric field. Moreover, they proposed a mathematical model based on Weibull equation with electric field strength and treatment time parameters to predict changes in anthocyanin content during HIPEF treatment [52]. Regardless of the type of bioactive component, in almost all cases (Figure 2), the maximum values of bioactive compounds were at highest value of electric field strength (50 kV cm^−1^), which is consistent with other literature data [48]. Better permeabilization is associated with an increase in tissue electrical conductivity due to HIPEF treatment [53]; therefore, an increase in cell disruption and ultimately better extraction of bioactive compounds has been observed with an increase in electric field strength [38,54].

When considering the influence of frequency on bioactive compounds, the lower frequency (100 Hz) had a positive effect on the content of individual bioactive compounds than the higher frequency (200 Hz). These results are confirmed by the research of Martin-Garcia et al. who performed an optimization of the HIPEF parameters of brewers’ spent grain (BSG): electric field strength (0.5, 1.5, and 2.5 kV cm^−1^), frequency (50, 100, and 150 Hz) and total treatment time (5, 10, and 15 s), where the maximum values of total polyphenolic compounds were at the highest value of electric field strength (2.5 kV cm^−1^) and the lowest frequency tested (50 Hz) [48]. A possible explanation for this trend could be that lower frequencies result in a higher degree of permeabilization of the plant tissue compared to higher frequencies, which consequently translates into better extraction of the respective compounds [55,56].

## 4. Conclusions

Strawberry juices from different ripening stages differed significantly in terms of physicochemical parameters and bioactive compound contents. Regarding the influence of HIPEF parameters on physicochemical properties of strawberry juice, no statistically significant changes in pH were observed, while higher electric field strength and longer treatment duration influenced the increase in SSC value.

In general, HIPEF-treated strawberry juices showed an 8.41% higher content of bioactive compounds compared to untreated juices, regardless of ripeness and processing parameters. Considering the influence of HIPEF parameters on the content of bioactive compounds, the increase in electric field strength led to an increase in the content of most bioactive compounds (except flavonols). Moreover, frequency, in contrast to electric field strength, showed an inverse effect on the content of all observed bioactive compounds. Treatment duration had no effect on the content of most observed bioactive compounds (except flavonols and condensed tannins). Storage at +4 °C for 7 days resulted in an increase in all phenols studied (except flavonols) and a decrease in SSC.

Based on the obtained results, the optimal parameters for carrying out the HIPEF treatment that affected the total phenolic content were as follows: electric field strength of 49.90 kV cm^−1^, frequency 199.74 Hz, treatment time 3.02 min with storage for 7 days. However, the optimization parameters indicated that the strawberry juices obtained at both ripening stages are suitable for HIPEF treatment, which can preserve the quality of strawberry juices.

## Figures and Tables

**Table 1:** Design of the experiment.

Sample	Juice	Storage (Days)	Treatment	Electric Field Strength (kV cm^−1^)	Frequency (Hz)	Pulse Duration (min)
1	J1	0	Control	/	/	/
2	J1	0	HIPEF	40	100	3
3	J1	0	HIPEF		200	3
4	J1	0	HIPEF		100	6
5	J1	0	HIPEF		200	6
6	J1	0	HIPEF	50	100	3
7	J1	0	HIPEF		200	3
8	J1	0	HIPEF		100	6
9	J1	0	HIPEF		200	6
10	J2	0	Control	/	/	/
11	J2	0	HIPEF	40	100	3
12	J2	0	HIPEF		200	3
13	J2	0	HIPEF		100	6
14	J2	0	HIPEF		200	6
15	J2	0	HIPEF	50	100	3
16	J2	0	HIPEF		200	3
17	J2	0	HIPEF		100	6
18	J2	0	HIPEF		200	6
19	J1	7	Control	/	/	/
20	J1	7	HIPEF	40	100	3
21	J1	7	HIPEF		200	3
22	J1	7	HIPEF		100	6
23	J1	7	HIPEF		200	6
24	J1	7	HIPEF	50	100	3
25	J1	7	HIPEF		200	3
26	J1	7	HIPEF		100	6
27	J1	7	HIPEF		200	6
28	J2	7	Control	/	/	/
29	J2	7	HIPEF	40	100	3
30	J2	7	HIPEF		200	3
31	J2	7	HIPEF		100	6
32	J2	7	HIPEF		200	6
33	J2	7	HIPEF	50	100	3
34	J2	7	HIPEF		200	3
35	J2	7	HIPEF		100	6
36	J2	7	HIPEF		200	6

J1—strawberry juice prepared from 75% ripe strawberries; J2—strawberry juice prepared from 100% ripe strawberries. Control—untreated samples.

**Table 2:** Changes in physicochemical parameters in untreated strawberry juices during storage.

Variables	n	SSC (%)	pH
Maturity		*p* ≤ 0.01 ^†^	*p* ≤ 0.01 ^†^
75% (Juice J1)	4	6.15 ± 0.03 ^b^	3.38 ± 0.01 ^b^
100% (Juice J2)	4	8.03 ± 0.03 ^a^	3.53 ± 0.01 ^a^
Storage	4	*p* ≤ 0.01 ^†^	*p* = 0.02 ^†^
0 days	4	7.20 ± 0.03 ^a^	3.48 ± 0.01 ^a^
7 days	8	6.98 ± 0.03 ^b^	3.43 ± 0.01 ^a^
Dataset average		7.09 ± 0.02	3.41 ± 0.01

Results are expressed as mean ± standard error. Values represented with different letters are statistically different at *p* ≤ 0.05; ^†^—significant factor in multifactor analysis; ^‡^—not significant factor in multifactor analysis. SSC—soluble solids content (%).

**Table 3:** Changes in physiochemical parameters in HIPEF-treated strawberry juices during storage.

Variables	n	SSC (%)	pH
Maturity		*p* ≤ 0.01 ^†^	*p* ≤ 0.01 ^†^
75% (Juice J1)	32	5.82 ± 0.01 ^b^	3.44 ± 0.01 ^b^
100% (Juice J2)	32	7.92 ± 0.01 ^a^	3.51 ± 0.01 ^a^
Storage		*p* ≤ 0.01 ^†^	*p* = 0.22 ^‡^
0 days	32	6.97 ± 0.01 ^a^	3.48 ± 0.01 ^a^
7 days	32	6.78 ± 0.01 ^b^	3.46 ± 0.01 ^a^
Electric field strength		*p* ≤ 0.01 ^†^	*p* = 0.41 ^‡^
40 kV cm^−1^	32	6.82 ± 0.01 ^b^	3.46 ± 0.01 ^a^
50 kV cm^−1^	32	6.92 ± 0.01 ^a^	3.48 ± 0.01 ^a^
Frequency		*p* = 0.39 ^‡^	*p* = 0.23 ^‡^
100 Hz	32	6.86 ± 0.01 ^a^	3.46 ± 0.01 ^a^
200 Hz	32	6.89 ± 0.01 ^a^	3.48 ± 0.01 ^a^
Treatment time		*p* = 0.31 ^‡^	*p* = 0.21 ^‡^
3 min	32	6.85 ± 0.01 ^a^	3.48 ± 0.01 ^a^
6 min	32	6.89 ± 0.01 ^a^	3.46 ± 0.01 ^a^
Dataset average	64	6.87 ± 0.01	3.47 ± 0.01

Results are expressed as mean ± standard error. Values represented with different letters are statistically different at *p* ≤ 0.05; ^†^—significant factor in multifactor analysis; ^‡^—not significant factor in multifactor analysis. SSC—soluble solids content (%).

**Table 4:** Changes in the contents of bioactive compounds in untreated strawberry juices during storage.

Variables	n	TPC	ANT	HCA	FL	CT
Maturity		*p* ≤ 0.01 ^†^	*p* ≤ 0.01 ^†^	*p* ≤ 0.01 ^†^	*p* ≤ 0.01 ^†^	*p* ≤ 0.01 ^†^
75% (Juice J1)	4	85.19 ± 1.77 ^a^	16.87 ± 0.20 ^b^	9.76 ± 0.44 ^b^	1.78 ± 0.10 ^b^	56.28 ± 0.58 ^b^
100% (Juice J2)	4	74.39 ± 1.77 ^b^	36.80 ± 0.20 ^a^	12.71 ± 0.44 ^a^	2.29 ± 0.10 ^a^	64.70 ± 0.58 ^a^
Storage		*p* ≤ 0.01 ^†^	*p* ≤ 0.01^†^	*p* ≤ 0.01 ^†^	*p* ≤ 0.01 ^†^	*p* ≤ 0.01 ^†^
0 days	4	63.55 ± 1.77 ^b^	23.82 ± 0.20 ^b^	8.76 ± 0.44 ^b^	2.94 ± 0.10 ^a^	41.20 ± 0.58 ^b^
7 days	4	96.02 ± 1.77 ^a^	29.85 ± 0.20 ^a^	13.71 ± 0.44 ^a^	1.13 ± 0.10 ^b^	79.78 ± 0.58 ^a^
Dataset average	8	79.78 ± 1.25	26.83 ± 0.14	11.24 ± 0.31	2.03 ± 0.07	60.49 ± 0.40

Results are expressed as mean ± standard error. Values represented with different letters are statistically different at *p* ≤ 0.05; ^†^—significant factor in multifactor analysis; ^‡^—not significant factor in multifactor analysis. ANT—anthocyanins (mg Pg-3-G 100 mL^−1^); TPC—total phenolic compounds (mg GAE 100 mL^−1^); HCA—hydroxycinnamic acids (mg CAE 100 mL^−1^); FL—flavonols (mg QE 100 mL^−1^); CT—condensed tannins (mg CA 100 mL^−1^).

**Table 5:** Changes in the contents of bioactive compounds in HIPEF-treated strawberry juices during storage.

Variables	n	TPC	ANT	HCA	FL	CT
Maturity		*p* ≤ 0.01 ^†^	*p* ≤ 0.01 ^†^	*p* ≤ 0.01 ^†^	*p* ≤ 0.01 ^†^	*p* ≤ 0.01 ^†^
75% (Juice J1)	32	96.91 ± 1.52 ^a^	19.01 ± 0.18 ^b^	10.62 ± 0.12 ^b^	1.81 ± 0.04 ^b^	64.99 ± 0.22 ^b^
100% (Juice J2)	32	82.08 ± 1.52 ^b^	37.97 ± 0.18 ^a^	14.10 ± 0.12 ^a^	2.48 ± 0.04 ^a^	69.99 ± 0.22 ^a^
Storage		*p* ≤ 0.01 ^†^	*p* ≤ 0.01 ^†^	*p* ≤ 0.01 ^†^	*p* ≤ 0.01 ^†^	*p* ≤ 0.01 ^†^
0 days	32	83.11 ± 1.52 ^b^	27.35 ± 0.18 ^b^	10.14 ± 0.12 ^b^	3.29 ± 0.04 ^a^	54.79 ± 0.22 ^b^
7 days	32	95.88 ± 1.52 ^a^	29.63 ± 0.18 ^a^	14.58 ± 0.12 ^a^	1.00 ± 0.04 ^b^	80.18 ± 0.22 ^a^
Electric field strength		*p* ≤ 0.01 ^†^	*p* ≤ 0.01 ^†^	*p* ≤ 0.01 ^†^	*p* ≤ 0.01 ^†^	*p* ≤ 0.01 ^†^
40 kV cm^−1^	32	85.89 ± 1.52 ^b^	27.73 ± 0.18 ^b^	11.59 ± 0.12 ^b^	1.91 ± 0.04 ^b^	63.98 ± 0.22 ^b^
50 kV cm^−1^	32	93.11 ± 1.52 ^a^	29.26 ± 0.18 ^a^	13.12 ± 0.12 ^a^	2.38 ± 0.04 ^a^	70.99 ± 0.22 ^a^
Frequency		*p* = 0.27 ^‡^	*p* = 0.03 ^†^	*p* ≤ 0.01 ^†^	*p* ≤ 0.01 ^†^	*p* ≤ 0.01 ^†^
100 Hz	32	90.69 ± 1.52 ^a^	28.79 ± 0.18 ^a^	12.60 ± 0.12 ^a^	2.29 ± 0.04 ^a^	68.57 ± 0.22 ^a^
200 Hz	32	88.31 ± 1.52 ^a^	28.19 ± 0.18 ^a^	12.12 ± 0.12 ^b^	2.00 ± 0.04 ^b^	66.40 ± 0.22 ^b^
Time		*p* = 0.99 ^‡^	*p* = 0.40 ^‡^	*p* = 0.30 ^‡^	*p* ≤ 0.01 ^†^	*p* = 0.05 ^†^
3 min	32	89.49 ± 1.52 ^a^	28.38 ± 0.18 ^a^	12.45 ± 0.12 ^a^	2.29 ± 0.04 ^a^	67.78 ± 0.22 ^a^
6 min	32	89.5 ± 1.52 ^a^	28.60 ± 0.18 ^a^	12.27 ± 0.12 ^a^	2.00 ± 0.04 ^b^	67.19 ± 0.22 ^b^
Dataset average	64	89.49 ± 0.50	28.60 ± 0.13	12.36 ± 0.09	2.15 ± 0.03	67.49 ± 0.14

Results are expressed as mean ± standard error. Values represented with different letters are statistically different at *p* ≤ 0.05; ^†^—significant factor in multifactor analysis; ^‡^—not significant factor in multifactor analysis. ANT—anthocyanins (mg Pg-3-G 100 mL^−1^); TPC—total phenolic compounds (mg GAE 100 mL^−1^); HCA—hydroxycinnamic acids (mg CAE 100 mL^−1^); FL—flavonols (mg QE 100 mL^−1^); CT—condensed tannins (mg CA 100 mL^−1^).

**Table 6:** Average values for experimental HIPEF settings.

Variables	T1 (°C)	T2 (°C)	ΔT (°C)	Voltage (kV)	Current (mA)	Power (W)
Electric field strength	*p* = 0.30 ^‡^	*p* = 0.21 ^‡^	*p* = 0.80 ^‡^	*p* ≤ 0.01 ^†^	*p* ≤ 0.01 ^†^	*p* ≤ 0.01 ^†^
40 kV cm^−1^	20.11 ± 1.04 ^a^	22.06 ± 0.57 ^a^	1.95 ± 1.42 ^a^	39.89 ± 0.02 ^b^	3.00 ± 0.02 ^b^	119.75 ± 0.72 ^b^
50 kV cm^−1^	21.75 ± 1.04 ^a^	23.16 ± 0.57 ^a^	1.41 ± 1.42 ^a^	49.93 ± 0.02 ^a^	3.84 ± 0.02 ^a^	190.00 ± 0.72 ^a^
Frequency	*p* = 0.32 ^‡^	*p* = 0.44 ^‡^	*p* = 0.30 ^‡^	*p* = 0.58 ^‡^	*p* ≤ 0.01 ^†^	*p* ≤ 0.01 ^†^
100 Hz	20.15 ± 1.04 ^a^	22.94 ± 0.57 ^a^	2.79 ± 1.42 ^a^	44.90 ± 0.02 ^a^	2.83 ± 0.02 ^b^	128.38 ± 0.72 ^b^
200 Hz	21.71 ± 1.04 ^a^	22.29 ± 0.57 ^a^	0.58 ± 1.42 ^a^	44.91 ± 0.02 ^a^	4.01 ± 0.02 ^a^	181.38 ± 0.72 ^a^
Treatment time	*p* = 0.50 ^‡^	*p* = 0.49 ^‡^	*p* = 0.82 ^‡^	*p* = 0.12 ^‡^	*p* = 0.58 ^‡^	*p* = 0.25 ^‡^
3 min	20.41 ± 1.04 ^a^	22.33 ± 0.57 ^a^	1.91 ± 1.42 ^a^	44.89 ± 0.02 ^a^	3.41 ± 0.02 ^a^	154.25 ± 0.72 ^a^
6 min	21.45 ± 1.04 ^a^	22.90 ± 0.57 ^a^	1.45 ± 1.42 ^a^	44.93 ± 0.02 ^a^	3.43 ± 0.02 ^a^	155.50 ± 0.72 ^a^
Dataset average	20.93 ± 0.74	22.93 ± 0.40	1.68 ± 1.00	44.91 ± 0.11	3.42 ± 0.01	154.88 ± 0.51

Results are expressed as mean ± standard error. Values represented with different letters are statistically different at *p* ≤ 0.05; ^†^—significant factor in multifactor analysis; ^‡^—not significant factor in multifactor analysis.

**Table 7:** Optimal HIPEF parameters for maximum mg 100 mL^−^^1^ of polyphenols in strawberry juices.

Analytical Variable	TPC	ANT	HCA	FL	CT
Content (mg 100 mL^−1^)	113.75	41.04	18.00	4.85	86.07
Maturity (%)	75.40	100.00	100.00	100.00	100.00
Storage (days)	6.96	7.00	7.00	0.00	7.00
Field (kV cm^−1^)	49.90	50.00	50.00	50.00	50.00
Frequency (Hz)	199.74	100.00	100.00	100.00	100.00
Time (min)	3.02	3.00	6.00	3.00	3.00

TPC—total phenolic compounds (mg GAE 100 mL^−^^1^); ANT—anthocyanins (mg Pg-3-G 100 mL^−^^1^); HCA—hydroxycinnamic acids (mg CAE 100 mL^−^^1^); FL—flavonols (mg QE 100 mL^−^^1^); CT—condensed tannins (mg CA 100 mL^−^^1^).

## Data Availability

Not applicable.
